# Pravastatin Chronic Treatment Sensitizes Hypercholesterolemic Mice Muscle to Mitochondrial Permeability Transition: Protection by Creatine or Coenzyme Q_10_

**DOI:** 10.3389/fphar.2017.00185

**Published:** 2017-04-05

**Authors:** Estela N. B. Busanello, Ana C. Marques, Noelia Lander, Diogo N. de Oliveira, Rodrigo R. Catharino, Helena C. F. Oliveira, Anibal E. Vercesi

**Affiliations:** ^1^Departamento de Patologia Clínica, Faculdade de Ciências Médicas, Universidade Estadual de CampinasSão Paulo, Brazil; ^2^Departamento de Biologia Estrutural e Funcional, Instituto de BiologiaUniversidade Estadual de Campinas, São Paulo, Brazil

**Keywords:** pravastatin, muscle mitochondria, mitochondrial permeability transition, catalase, LDL receptor knockout mice

## Abstract

Statins are efficient cholesterol-lowering medicines utilized worldwide. However, 10% of patients suffer from adverse effects specially related to skeletal muscle function. Pro- or anti-oxidant effects of statins have been reported. Here we hypothesized that statins induce muscle mitochondrial oxidative stress leading to mitochondrial permeability transition (MPT) which may explain statin muscle toxicity. Thus, our aims were to investigate the effects of statin chronic treatment on muscle mitochondrial respiration rates, MPT and redox state indicators in the context of hypercholesterolemia. For this purpose, we studied muscle biopsies of the hypercholesterolemic LDL receptor knockout mice (*LDLr^-/-^)* treated with pravastatin during 3 months. Plantaris, but not soleus muscle of treated mice showed significant inhibition of respiration rates induced by ADP (–14%), oligomycin (–20%) or FCCP (–40%). Inhibitions of respiratory rates were sensitive to EGTA (Ca^2+^ chelator), cyclosporin A (MPT inhibitor), ruthenium red (inhibitor of mitochondria Ca^2+^ uptake) and coenzyme Q_10_ (antioxidant), indicating that pravastatin treatment favors Ca^2+^ induced MPT. Diet supplementation with creatine (antioxidant) also protected treated mice against pravastatin sensitization to Ca^2+^ induced MPT. Among several antioxidant enzymes analyzed, only catalase activity was increased by 30% in plantaris muscle of pravastatin treated mice. Oxidized lipids, but not proteins biomarkers were identified in treated *LDLr^-/-^* plantaris muscle. Taken together, the present results suggest that chronic pravastatin administration to a model of familial hypercholesterolemia promotes mitochondrial dysfunctions in plantaris muscle that can be counteracted by antioxidants administered either *in vitro* (CoQ_10_) or *in vivo* (creatine). Therefore, we propose that inhibition of muscle mitochondrial respiration by pravastatin leads to an oxidative stress that, in the presence of calcium, opens the permeability transition pore. This mitochondrial oxidative stress caused by statin treatment also signals for cellular antioxidant system responses such as catalase upregulation. These results suggest that the detrimental effects of statins on muscle mitochondria could be prevented by co-administration of a safe antioxidant such as creatine or CoQ10.

## Introduction

Statins are fungal-derived or synthetic cholesterol-lowering medicines that act by inhibiting 3-hydroxy-3-methylglutaryl coenzyme-A (HMG-CoA) reductase, the rate-limiting enzyme in cholesterol synthesis (Endo, 1992; Tobert, 2003). These medicines are the most commonly prescribed worldwide and represent the primary treatment strategy for hypercholesterolemia and prevention of mortality related to atherosclerosis (Naci et al., 2013). In addition to lowering plasma cholesterol levels, statins are claimed to exhibit pleiotropic effects that include an antioxidant action (Carneado et al., 2002; Wassmann et al., 2002; Manfredini et al., 2010). Therefore, it has been suggested that statins could also have beneficial effects in the treatment of oxidative stress associated diseases such as metabolic syndrome, sepsis, neurological conditions and even tumors (Dobesh and Olsen, 2014; Malfitano et al., 2014; Vallianou et al., 2014). On the other hand, approximately 10% of the patients under statin treatment develop a variety of muscle symptoms including myalgia, muscle cramps, and rarely rhabdomyolysis (Bruckert et al., 2005).

While inhibiting cholesterol synthesis, statins also inhibit the production of ubiquinone (CoQ_10_) and other intermediaries including dolichol and isoprenoids (Sirvent et al., 2008). CoQ_10_ is a component of the electron transport chain and also displays antioxidant properties in its reduced form (ubiquinol). Although the molecular mechanisms underlying statin-induced myotoxicity are not yet fully understood, a common hypothesis suggests that it is mediated by inhibition of mitochondrial respiration as a consequence of CoQ_10_ depletion (Päivä et al., 2005; Bookstaver et al., 2012; Larsen et al., 2013). In addition, previous studies propose that statins cause cell death associated with alterations in calcium homeostasis, inhibition of beta-oxidation, inhibition of mitochondrial respiratory complexes I and II followed by mitochondrial oxidative stress (Kaufmann et al., 2006; Oliveira et al., 2008; Costa et al., 2013; La Guardia et al., 2013) and also inhibition of complex III (Schirris et al., 2015). We have previously shown that statins stimulate Ca^2+^ induced mitochondrial permeability transition (MPT) in mitochondria isolated from murine liver and muscle, and from mice treated with lovastatin (Velho et al., 2006). Ca^2+^ and reactive oxygen species (ROS) act synergistically in the mechanism of MPT, a non-specific permeabilization of the inner mitochondrial membrane that (Kowaltowski et al., 2000) triggers cell death under a variety of pathological conditions or drug toxicity (Vercesi et al., 2006; Rasola and Bernardi, 2011; Javadov and Kuznetsov, 2013). The close localization of mitochondria and the endoplasmic reticulum (ER) in situ (Hajnóczky and Csordás, 2010) allows for rapid Ca^2+^ uptake by mitochondria from the ER microdomains. The existence of a redox controlled cross talk between mitochondria and the ER involving NADPH oxidases has been described (Dikalov, 2011). These redox interactions may control MPT and the execution of Ca^2+^ signaling for cell death (Figueira et al., 2013).

Sacher et al. (2005) reported that simvastatin and lovastatin activate the mitochondrial pathway of apoptosis in primary cultures of human skeletal muscle obtained from healthy individuals. We have further investigated the mechanisms of cell death induced by simvastatin in PC3 prostate cancer cells that underwent necrosis, in a manner sensitive to cyclosporine A (CsA), an MPT inhibitor. The necrotic cell death was preceded by increased cytosolic free Ca^2+^ concentration, ROS generation, inhibition of respiration and mitochondrial membrane potential disruption (Oliveira et al., 2008). Kwak et al. (2012) showed that simvastatin impairs ADP-stimulated respiration at the level of complex I, increases ROS generation and induces apoptosis in human skeletal muscle primary culture. We have also shown that in rat soleus muscle fibers incubated with simvastatin, the content of CoQ_10_ was reduced by 40% and addition of CoQ_10_ in these muscle biopsies prevented the inhibition of respiration at complex I and II levels and MPT, via free radical scavenging properties (Deichmann et al., 2010; La Guardia et al., 2013). Therefore, findings regarding statins redox effects are controversial and include antioxidant (Carneado et al., 2002; Wassmann et al., 2002; Manfredini et al., 2010; Zhou and Liao, 2010) and pro-oxidant actions (Velho et al., 2006; Oliveira et al., 2008; Kwak et al., 2012; La Guardia et al., 2013). In line with our previous works, here we hypothesized that statins induce muscle mitochondrial oxidative stress, which increases susceptibility to MPT. Thus, our aims were to investigate the effects of statin chronic treatment on muscle mitochondrial respiration rates, MPT and redox state indicators in the context of hypercholesterolemia. For this purpose, we used the mouse model that mimics the human disease familial hypercholesterolemia, since statins are used to treat specifically genetic hypercholesterolemic patients. We also chose a therapeutic dose of a hydrophilic statin (pravastatin) and two types of muscles predominantly aerobic (soleus) or anaerobic (plantaris) as target tissues.

## Materials and Methods

### Animals and Reagents

LDL receptor knockout mice (*LDLr^-/-^*) founders were purchased from Jackson Laboratory (Bar Harbor, ME) and the breeding colony was maintained at the Universidade Estadual de Campinas (CEMIB-Unicamp), Campinas, Brazil. *LDLr^-/-^* mice had access to standard laboratory rodent chow (AIN 93M, PragSoluções, SP, Brazil), and water *ad libitum* and were housed at 22 ± 2°C on a 12h light-dark cycle. This study was performed in accordance with the Guide for the Care and Use of Laboratory Animals published by National Academy of Sciences and with the approval of University Committee for Ethics in Animal Experimentation (protocol # 3401-1). Chemicals were purchased from Sigma (St. Louis, MO, USA).

### Pravastatin Treatment and Creatine Supplementation

Thirty-day-old male *LDLr^-/-^* mice received pravastatin sodium (Medley) diluted in the drinking water (400 mg/L) during 2 or 3 months according to Lorza-Gil et al. (2016). The estimated pravastatin dose of 40 mg/Kg body weight per day was based on average consumption rate measurements (3.5 mL/day). Controls received filtered tap water without pravastatin. Additional groups of mice were treated with 2% creatine supplemented into standard diet (AIN 93M, PragSoluções, SP, Brazil) without alteration of total calories during the last 15 days of pravastatin treatment.

### Plasma Cholesterol Analysis

Blood samples were collected with heparin from *LDLr^-/-^* mice tail between 8 and 9 am after a 12-h fasting. Samples were centrifuged and plasma was utilized for cholesterol measurement using a standard commercial kit (Roche Diagnostics) according to the manufacturer’s instructions. Plasma cholesterol levels in pravastatin treated *LDLr^-/-^* mice were significantly reduced compared to untreated mice (437 ± 57 vs. 390 ± 36, respectively, *P* < 0.05).

### Skeletal Muscle Sample Preparation

Plantaris and soleus muscles were harvested from *LDLr^-/-^* mice and placed on ice-cold buffer containing 10 mM Ca-EGTA buffer (2.77 mM of CaK_2_EGTA + 7.23 mM of K_2_EGTA, free concentration of calcium 0.1 mmol/L), 20 mmol/L imidazole, 50 mmol/L K^+^/ 4-morpholinoethanesulfonic acid, 0.5 mmol/L dithiothreitol, 7 mmol/L MgCl_2_, 5 mmol/L ATP, 15 mmol/L phosphocreatine, pH 7.1. Individual fiber bundles from three to 5 mg of soleus or plantaris skeletal muscle were separated with forceps. Samples were permeabilized in ice-cold buffer containing saponin (50 μg/mL) during 30 min, gently stirred and washed three times with MiR05 medium (60 mmol/L potassium lactobionate, 0.5 mmol/L EGTA, 3 mmol/L MgCl_2_, 20 mmol/L taurine, 10 mmol/L KH_2_PO_4_, 20 mmol/L HEPES, 110 mmol/L sucrose, 1 g/L BSA, pH 7.1) at 4°C. Samples were dried with filter paper and weighted (Kuznetsov et al., 2008; La Guardia et al., 2013).

### Oxygen Consumption

**FIGURE 1:**
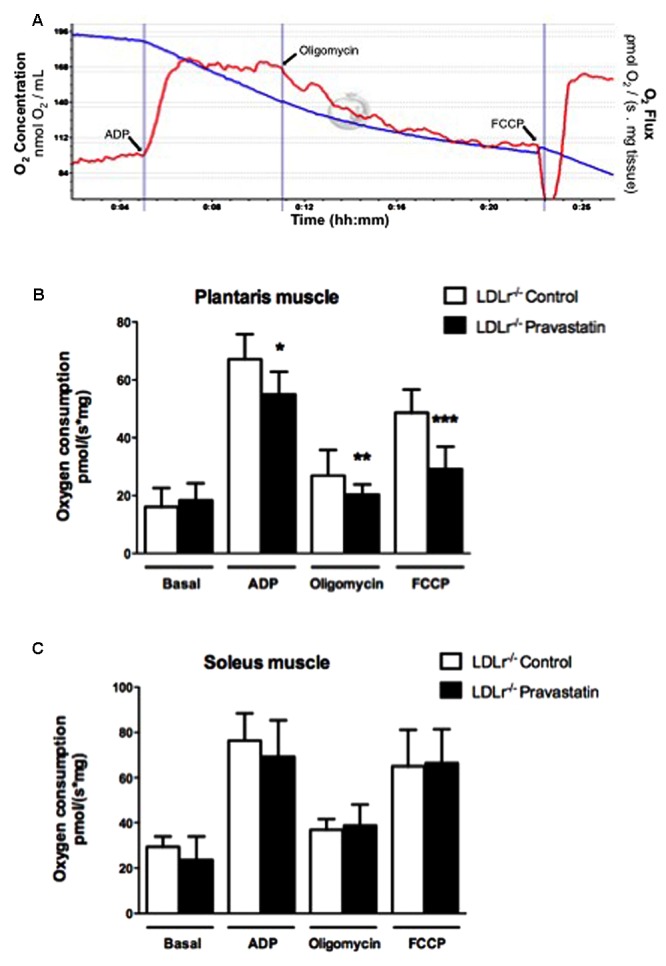
**Pravastatin treatment inhibits oxygen consumption by plantaris muscle from *LDLr^-/-^* mice in the presence of Ca^2+^.** Respiration was evaluated in a medium MiR05 at 37°C containing 10 mM glutamate plus 5 mM malate as substrates in the presence of Ca^2+^ (4.4 μM). ADP (400 μM), oligomycin (0.63 μM), and FCCP (0.6 μM) were added during the experiments. Representative traces of plantaris respiration where O_2_ concentration (blue line) is expressed as nmol O_2_/mL and O_2_ flux per mass (red line) is expressed as ρmol O_2_/s. mg tissue **(A)**. Bar graphs show plantaris **(B)** and soleus muscle **(C)** from *LDLr^-/-^* mice treated or not with pravastatin (40 mg/Kg/day). Values are means ± standard deviation and are expressed as ρmol O_2_/s. mg tissue. ^∗^*P* = 0.0103, ^∗∗^*P* = 0.0442, ^∗∗∗^*P* = 0.0004 compared to control (Student’s *t*-test). *N* = 7–9, at least seven independent experiments.

Oxygen consumption was evaluated in permeabilized skeletal muscle according to Kuznetsov et al. (2008) and La Guardia et al. (2013) with slight modifications. Permeabilized tissues were added to MiR05 medium without EGTA containing Ca^2+^ (4.4 μM) at 37°C supported with 10 mM glutamate plus 5 mM malate in a high-resolution oxygraph OROBOROS (Innsbruck, Austria). ADP (400 μM), oligomycin (0.63 μM), and FCCP (0.6 μM) were added during the experiments. Some analyses were evaluated in the presence of EGTA (500 μM), CsA (0.83 mM), ruthenium red (1 μM) or coenzyme Q_10_ (10 μM). **Figure 1A** shows the typical experimental respiratory profile.

### Tissue Preparation and Enzymatic Activities

Plantaris and soleus muscles were harvested from *LDLr^-/-^* mice and homogenized in 9 volumes (1:10, w/v) of 20 mM sodium phosphate buffer, pH 7.4 containing 140 mM KCl. Homogenates were centrifuged at 1000 × *g* for 10 min at 4°C for nuclei and cell debris removal (Evelson et al., 2001). The pellet was discarded and the total supernatant was used for enzymatic activity determination.

Glutathione peroxidase, glutathione reductase, superoxide dismutase and peroxiredoxin were determined according to Wendel (1981), Carlberg and Mannervik (1985), Marklund (1985), and Kim et al. (2005), respectively. Catalase activity was analyzed by measuring the absorbance decrease at 240 nm according to Aebi (1984) and one unit (U) of the enzyme is defined as the metabolization of 1 μmol of H_2_O_2_ per min. The specific activity was calculated and expressed as U/mg protein. The activity of aconitase was measured according to Morrison (1954), following the reduction of NADP^+^ at wavelengths of excitation and emission of 340 and 466 nm, respectively. Aconitase activity was expressed as nmol NADPH/min/mg protein. Protein content was measured according to Lowry et al. (1951) using bovine serum albumin as standard.

### Reverse Transcriptase (RT)-qPCR

Catalase mRNA expression was quantified by RT-qPCR using GAPDH housekeeping gene to normalize each sample. Plantaris muscles were harvested from *LDLr^-/-^* mice, total RNA was extracted using TRIzol (Thermo Fisher Scientific) following manufacturer’s instructions. Total RNA was used as template for cDNA synthesis in a reaction with oligo(dT)_18_ primer (Exxtend Biotecnologia) and SuperScript III Reverse Transcriptase (Thermo Fisher Scientific) at 50°C for 60 min. The enzyme was then inactivated at 70°C for 15 min. Real-time PCR was performed on a Rotor Gene system (Qiagen, Hilden, Germany) using Rotor Gene SYBR Green PCR kit (Qiagen) and the following cycling conditions: 95°C for 5 s and 60°C for 10 s. Data acquisition was performed during the annealing step at 60°C. Primers used in qPCR were as follows: *CAT* (98 bp), 5′ GTTGAACGAGGAGGAGAGG 3′ (forward) and 3′ GTGAAATTCTTGACCGCTTTC 5′ (reverse); *GAPDH* (175 bp), 5′ GCACCACCAACTGCTTAGC 3′ (forward) and 3′ ATGCAGGGATGATGTTCTGG 5′ (reverse). *CAT* and *GAPDH* mRNA quantification was performed twice in *N* = 5 animals from each group. Data were analyzed using the Delta CT method of Rotor Gene Q series Software and catalase relative mRNA expression levels were obtained by normalizing against the level of *GAPDH* from the same sample and conditions. Efficiencies of *CAT* and *GAPDH* qPCRs were 1.00 and 0.90, respectively. Standard curves were prepared for each run using known quantities of pGEM-T-easy plasmids (Promega) containing *CAT* and *GAPDH* genes.

### Sulfhydryl Content

Protein oxidative damage was evaluated by sulfhydryl content measurement according to Aksenov and Markesbery (2001). The reduction of 5,5′-dithio-bis (2-nitrobenzoic acid (DTNB) by thiols present in the sample generates a yellow compound (TNB) whose absorption is measured spectrophotometrically at 412 nm. Briefly, 30 μL of 10 mM DTNB and 980 μL of PBS were added to 50 μL muscle supernatant followed by a 30 min incubation at room temperature in the dark. The absorption measured was proportional to the amount of thiol groups present in the sample. Results were calculated as nmol TNB/mg of protein.

### Electrospray Ionization High Resolution Mass Spectrometry (ESI-HRMS) Analysis

Plantaris muscles were removed from *LDLr^-/-^* mice and rapidly homogenized with a methanol:H_2_O (50:50) solution under sonication. Resulting homogenates were filtered through a 0.22 μm nylon membrane; 10 μL of the filtrate were further diluted in methanol:H_2_O (50:50) solution containing 0.1% formic acid to a final volume of 1 mL. Samples were directly infused in an ESI-LTQ-XL Orbitrap Discovery instrument (Thermo Scientific, Bremen, Germany). Typical operating conditions were as follows: sheath gas at 10 arbitrary units, 4.5 kV and m/z range of 50–1000 in the positive ion mode. Structural elucidation was carried out using mass accuracy as the main parameter, with a mass shift (error) less than 2 ppm. Spectral data were submitted to a partial least squares discriminant analysis (PLS-DA) using MetaboAnalyst 3.0 (Xia et al., 2015) to identify markers for each condition. Data normalization was performed using log transformation and range scaling. The selected ions were then researched in the Lipid Maps database, where oxidized species were identified.

### Statistical Analysis

Results are presented as mean ± standard deviation of at least eight mice Data were analyzed using one-way analysis of variance (ANOVA) followed by the *post hoc* Tukey’s multiple comparison test when *F* was significant. The Student’s *t-*test for unpaired samples was also used for two-means comparisons. Differences between groups were rated significant at *P* < 0.05. All analyses were carried out using the GraphPad software.

## Results

### Inhibition of Respiration Supported by Site I Substrates in Plantaris Muscle Biopsies from LDLr^-/-^ Mice Treated with Pravastatin

In order to investigate the effects of pravastatin chronic treatment on mitochondrial respiration of soleus and plantaris muscle, *LDLr^-/-^* mice received pravastatin (40 mg/kg/day) added to the drinking water during 3 months. Oxygen consumption supported by 10 mM glutamate plus 5 mM malate was evaluated in the presence of Ca^2+^ (4.4 μM) with the addition of ADP (400 μM), oligomycin (0.63 μM), and FCCP (0.6 μM) during the experiments. **Figure 1A** shows typical traces of mitochondrial respiration rates in all conditions. **Figure 1B** shows that pravastatin treatment promoted significant inhibition of mitochondrial respiration in all states: phosphorylating (ADP), resting (oligomycin) and maximal (FCCP) respiration rates of plantaris muscle in the presence of Ca^2+^. The inhibitions were 14, 24, and 40% for ADP-, oligomycin- and FCCP- stimulated respiration, respectively [*n* = 8; *P* = 0.0103; *P* = 0.0442; *P* = 0.0004]. The lower rate of FCCP-induced respiration compared to ADP-induced respiration is in agreement with recent data (Ruas et al., 2016) showing that oligomycin treatment previous to FCCP addition leads to an underestimation of maximal respiratory capacity induced by FCCP. In contrast to plantaris, no significant alterations of oxygen consumption rates were observed in soleus muscle (**Figure 1C**). Furthermore, no differences in oxygen consumption were observed in plantaris after only 2 months of pravastatin treatment (data not shown).

### Pravastatin-Induced Inhibition of Mitochondrial Respiration Is Dependent on Mitochondrial Permeability Transition Pore (PTP) Opening

**FIGURE 2:**
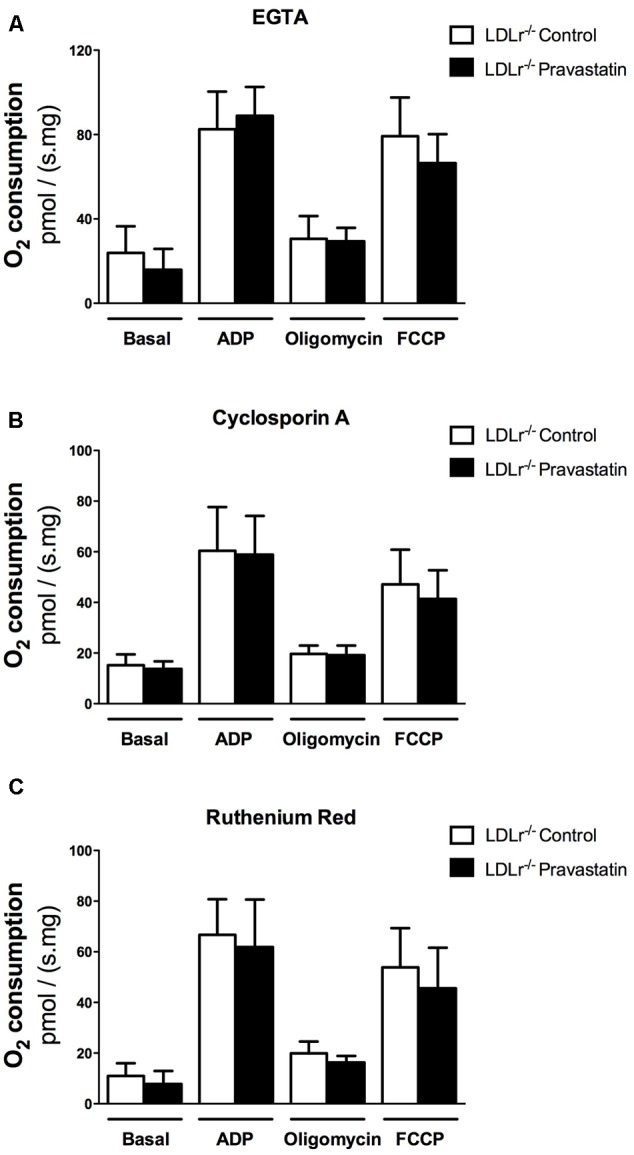
**Pravastatin treatment does not inhibit oxygen consumption in the presence of EGTA, cyclosporin A or ruthenium red in permeabilized plantaris muscle of *LDLr^-/-^* mice.** Respiration was evaluated in a medium MiR05 at 37°C containing 10 mM glutamate plus 5 mM malate as substrates in the presence of 500 μM EGTA **(A)**, 0.83 mM cyclosporin A **(B)** or 1 μM ruthenium red **(C)** in plantaris muscle from *LDLr^-/-^* mice treated or not with pravastatin (40 mg/Kg/day). ADP (400 μM), oligomycin (0.63 μM) and FCCP (0.6 μM) were added during the experiments. Values are means ± standard deviation and are expressed as ρmol O_2_/s. mg tissue. No significant difference was observed. *N* = 10–12, at least ten independent experiments.

Considering that Ca^2+^ is essential for PTP opening (Hunter et al., 1976; Kowaltowski et al., 2001), and that toxic effects of statins have been associated with alterations in calcium homeostasis (Sirvent et al., 2005b; Sirvent et al., 2012), our next step was to investigate the role of Ca^2+^ on oxygen consumption of plantaris muscle of *LDLr^-/-^* mice. For this purpose, the Ca^2+^ chelator EGTA, ruthenium red (a mitochondrial Ca^2+^ uptake inhibitor) or cyclosporin A (CsA, a permeability transition inhibitor) were added in the reaction medium before oxygen consumption measurements. **Figure 2** shows that all these compounds fully reversed the mitochondrial respiration inhibition in plantaris muscle of pravastatin treated *LDLr^-/-^* mice.

### Both Creatine and Coenzyme Q_10_ Prevented Mitochondrial Respiratory Inhibition Induced by Pravastatin

**FIGURE 3:**
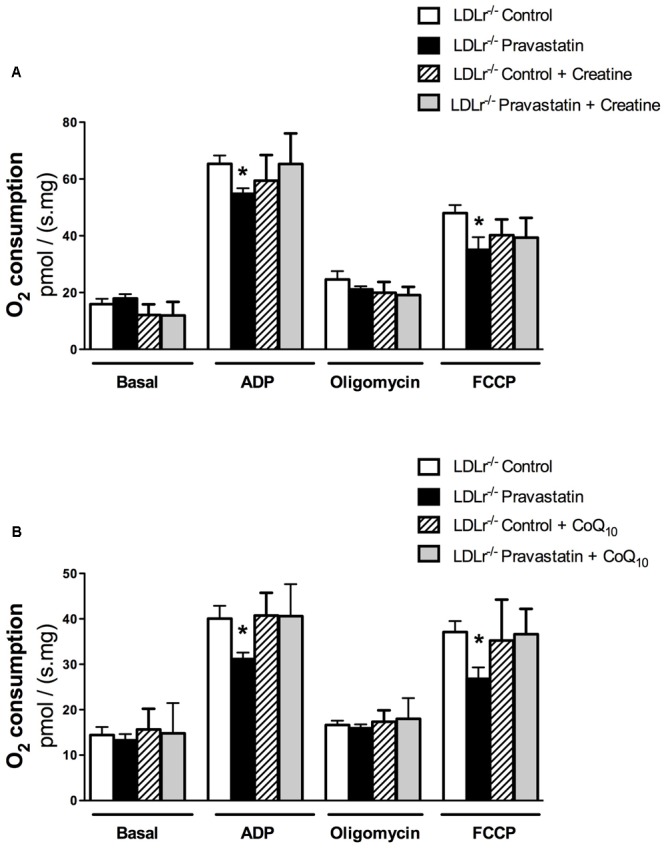
**Inhibition of oxygen consumption in the presence of Ca^2+^ is prevented by creatine (A)** or Coenzyme Q_10_ **(B)** in plantaris muscle of *LDLr^-/-^* mice treated with pravastatin (40 mg/kg/day). Respiration was evaluated in a medium MiR05 at 37°C containing 10 mM glutamate plus 5 mM malate as substrates in the presence of Ca^2+^ (4.4 μM). ADP (400 μM), oligomycin (0.63 μM), and FCCP (0.6 μM) were added during the experiments. Coenzyme Q_10_ (CoQ_10_, 10 μM) was added in the reaction medium before the biopsies. Values are means ± standard deviation and are expressed as ρmol O_2_/s. mg tissue. ^∗^*P* < 0.05 compared to control (One-Way ANOVA). *N* = 9–12, at least nine independent experiments.

Creatine acts directly as antioxidant (Lawler et al., 2002). In addition, creatine supplementation acts on ATP/ADP ratio maintenance due to creatine kinase (CK) activation and CK is part of the protein complex that is involved in MPT regulation (Kowaltowski et al., 2001; Dolder et al., 2003; Meyer et al., 2006). Therefore, we supplemented *LDLr^-/-^* mouse chow diet with 2% of creatine during the last 15 days of pravastatin treatment. **Figure 3A** shows that creatine diet supplementation prevented the inhibitory action of pravastatin on ADP- and FCCP-stimulated oxygen consumption in the presence of Ca^2+^ in plantaris muscle of *LDLr^-/-^* mice [*n* = 10; *P* < 0.05].

CoQ_10_, which was previously reported by us to protect against mitochondrial dysfunction caused by simvastatin in rat soleus muscle (La Guardia et al., 2013), also showed the same protective effect under the experimental *in vitro* conditions of mouse mitochondrial phosphorylation (ADP) and maximal respiration (FCCP) rates [*n* = 10; *P* < 0.05] (**Figure 3B**).

### Pravastatin Treatment Upregulates Catalase Activity and Induces Lipid Oxidation in Plantaris Muscle

**FIGURE 4:**
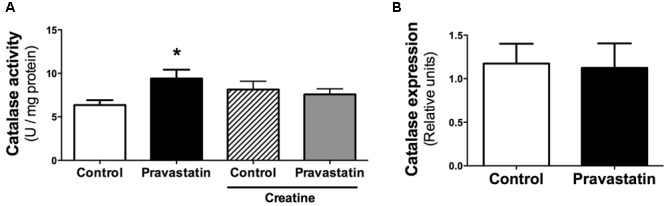
**Catalase activity (A)** and gene expression **(B)** evaluated in plantaris muscle of *LDLr^-/-^* mice. Plantaris muscles of control- and pravastatin-treated (40 mg/kg/day) *LDLr^-/-^* mice and both groups with creatine diet supplementation were used for catalase activity. Values are means ± standard deviation and are expressed in U/mg protein for activity and normalized by GAPDH for mRNA levels. ^∗^*P* < 0.05 compared to control (One-Way ANOVA and Student’s *t*-test). *N* = 12 for catalase activity and *N* = 5 for mRNA expression.

Considering that several studies claim an antioxidant activity of statins due to upregulation of antioxidant defenses (Carneado et al., 2002; Wassmann et al., 2002; Manfredini et al., 2010; Zhou and Liao, 2010), we investigated the activity of antioxidant enzymes in muscle of *LDLr^-/-^* mice. **Figure 4A** shows that pravastatin treatment increased catalase activity up to 30% in plantaris muscle homogenates. In addition, creatine diet supplementation abolished the differences in catalase activity between control and pravastatin treated mice [*n* = 5, *P* < 0.05]. This increase is probably the consequence of a pravastatin effect at a post-transcriptional step or on the enzymatic catalysis, since catalase mRNA expression levels were not altered in *LDLr^-/-^* mice muscle (**Figure 4B**). On the other hand, pravastatin treatment caused no differences in superoxide dismutase, glutathione reductase, glutathione peroxidase, peroxiredoxin and glucose-6-phosphate dehydrogenase activities either in plantaris or in soleus muscle (data not shown).

To verify that ROS production may occur due to pravastatin treatment, we investigated the presence of oxidized lipids in *LDLr^-/-^* mice. Using electrospray ionization high-resolution mass spectrometry analysis and a lipidomics approach, we identified oxidized lipid markers, especially phosphatidic acid and derivatives of arachidonic acid in plantaris muscle of *LDLr^-/-^* mice under pravastatin treatment (**Table 1**).

**Table 1:** Lipid markers identified by electrospray ionization high-resolution mass spectrometry in *LDLr^-/-^* mice.

			[M+Na]^+^		
	Lipid Class	Molecule	Theoretical Mass	Experimental Mass	Error (ppm)
**Control**	Glycerophosphoglycerol	PG (12:1) + O	465.1860	465.1869	1.9
	Prostaglandin D2	PGD2-G	449.2510	449.2508	–0.4
	Prostaglandin D2	1a,1b-dihomo-PGD2	403.2455	403.2460	1.2
	*N*-acyl amine	*N*-arachidonoyl (iso)leucine	440.3135	440.3126	–2.0
	Prostaglandin A2	PGA2 methyl ester, 15-acetate	413.2298	413.2292	–1.5
**Pravastatin**	Phosphatidic acid	PA (22:1) + O_2_	546.2934	546.2937	0.5
	Lyso-phosphoethanolamine	LysoPE (0:0/22:4)	552.3061	552.3072	2.0
	*N*-acyl amine	Arachidonoyl serotonin	485.3138	485.3132	–1.2
	Fatty acyl carnitine	O-arachidonoylcarnitine + O_2_	502.3139	502.313	–1.8
	Unsaturated fatty acid	C34:4+O_2_	555.4384	555.4381	–0.5

*Structure assignment was based on mass accuracy of ions elected by PLS-DA analysis. The experimental mass is obtained for the ionized molecule ([M+Na]^+^) and compared to the closest mass listed in the lipid map database LMID (theoretical mass). PLS-DA: Partial Least Squares Discriminant Analysis; Ppm: parts per million. Six independent experiments.*

To further investigate possible oxidative damage on other cellular components, we evaluated aconitase activity, a ROS-susceptible enzyme (Tretter and Adam-Vizi, 2000), and total sulfhydryl content, a protein oxidative damage marker in plantaris muscle of *LDLr^-/-^* mice. Both oxidative stress markers were not altered, suggesting that oxidative damage to proteins is probably not occurring in plantaris muscle of *LDLr^-/-^* mice under pravastatin treatment (**Supplementary Figure S1**).

## Discussion

Most literature data on statins toxicity indicate a series of metabolic alterations, such as inhibition of mitochondrial respiration (Kwak et al., 2012; La Guardia et al., 2013), imbalance in calcium homeostasis (Sirvent et al., 2005a; Oliveira et al., 2008), inhibition of β -oxidation (Kaufmann et al., 2006; Costa et al., 2013) and mitochondrial oxidative stress (Velho et al., 2006; Oliveira et al., 2008; Kwak et al., 2012; Abdoli et al., 2013; Costa et al., 2013; La Guardia et al., 2013). However, these data were obtained in normocholesterolemic wild type models or in cultured cells or isolated mitochondria. Here, we investigated the mechanisms underlying mitochondrial dysfunction and MPT in skeletal muscle biopsies of a familial hypercholesterolemic mice model under chronic treatment with therapeutic doses of the hydrophilic pravastatin. The present work provides evidence that plantaris (but not soleus) muscle from *LDLr^-/-^* mice treated during 3 months (but not less) with pravastatin presents both inhibition of respiration (40% reduction in maximal respiration rate) and MPT when Ca^2+^ is present in the incubation medium, a condition that may lead to cell death. The protection from these toxic statin effects by the antioxidants CoQ_10_ and creatine suggests the participation of ROS in this mechanism, in agreement with previous data (Velho et al., 2006; Manfredini et al., 2010; Abdoli et al., 2013; La Guardia et al., 2013).

Searching for possible oxidative damage signals, several oxidized lipids species were identified in mitochondria of pravastatin treated *LDLr^-/-^* plantaris muscle, reinforcing the existence of an oxidative insult. However, since no protein oxidation markers (diminished SH- groups content or aconitase activity) were found, we may conclude that the nature of this oxidative insult must be mild and/or partially counteracted by cell defenses. Upregulation of catalase activity in pravastatin treated *LDLr^-/-^* plantaris muscle is one of these cell defense responses to oxidative stress (Kirkman and Gaetani, 2007; Jackson and McArdle, 2011). This suggests the participation of a signaling pathway linking mild mitochondrial oxidative stress to activation of catalase (Zarse et al., 2012; Ristow, 2014). Indeed, it was previously shown that the antioxidant effects of statins are possibly related to their ability to upregulate antioxidant defenses, including catalase expression and activity *in vitro* and *in vivo* (Carneado et al., 2002; Wassmann et al., 2002; Manfredini et al., 2010). The minor oxidative signs observed in pravastatin treated *LDLr^-/-^* are in line with this homeostatic antioxidant response to a chronic and mild oxidative stress. This is also in accordance with the safety of these drugs and the fact that only 10% of statin-treated hypercholesterolemic patients present adverse effects (Bruckert et al., 2005).

Among the several oxidized lipids found in muscle of pravastatin treated mice, we highlight two species, phosphatidic acid and arachidonic acid derivatives. Phosphatidic acid acts as second messenger that regulates several proteins (Testerink and Munnik, 2005), including mTOR (mammalian target of rapamycin). It is required for the stability and activity of this protein kinase (Steed and Chow, 2001; Foster et al., 2014; Shad et al., 2015; Yoon et al., 2015; Ghim et al., 2016). Thus, we could speculate that the oxidation of phosphatidic acid caused by pravastatin may impair mTOR pathway, affecting the maintenance of muscle mass and protein turnover (Shad et al., 2015). On the other hand, arachidonic acid metabolites, such as prostaglandin and leukotriene are involved in inflammatory muscle pain, and also in myogenesis and muscle repair (Korotkova and Lundberg, 2014). Therefore, oxidized derivatives of arachidonic acid could also impair muscle repair process in *LDLr^-/-^* mice under pravastatin treatment.

Previous studies proposed that statin-induced myotoxicity may be mediated by the reduction of ubiquinone content (Sirvent et al., 2008). Accordingly, inhibition of mitochondrial respiration was associated with ubiquinone depletion (Päivä et al., 2005; Bookstaver et al., 2012; Larsen et al., 2013) and ubiquinol treatment protected human rhabdomyosarcoma cells against simvastatin-induced mitochondrial dysfunction and cell death (Vaughan et al., 2013). While several studies propose that ubiquinone depletion by statins may be deleterious due to impairment of mitochondrial respiration (Päivä et al., 2005; Bookstaver et al., 2012; Larsen et al., 2013; Vaughan et al., 2013), we previously provided evidence that the decreased levels of CoQ_10_ by statin are not enough to limit mitochondrial respiration but rather impair its free radical scavenger action leading to oxidative stress (La Guardia et al., 2013). In addition, the rate of hydrogen peroxide production was increased in the presence of simvastatin and was normalized by CoQ_10_, reinforcing the involvement of oxidative stress in simvastatin-induced toxicity to skeletal muscle (La Guardia et al., 2013).

Creatine supplementation, widely and safely used by athletes, exerts beneficial effects on muscle growth and strength as well as in rehabilitation (Hespel and Derave, 2007; D’Antona et al., 2014). Creatine also has direct antioxidant properties (Lawler et al., 2002; Sestili et al., 2006), inhibits PTP opening and reduces muscle necrosis (O’Gorman et al., 1996; Dolder et al., 2003). Based on these findings, we evaluated whether creatine diet supplementation would prevent pravastatin-induced myotoxicity. Indeed, creatine treatment reversed mitochondrial dysfunction of plantaris muscle of *LDLr^-/-^* mice.

An important finding of the present work is that the mitochondrial respiratory inhibition provoked by chronic pravastatin treatment was sensitive to Ca^2+^ chelator (EGTA), ruthenium red (an inhibitor of Ca^2+^ uptake by mitochondria) or CsA (MPT inhibitor). Therefore, mitochondrial permeability transition may explain the occurrence of muscle dysfunctions in patients sensitive to statin toxicity.

It is of note that these pravastatin effects on plantaris muscle were not observed in soleus muscle under the same experimental conditions. These distinct skeletal muscles present different types of metabolism and fiber composition. Plantaris is mainly composed by type II fibers, presenting less mitochondrial content and higher glycolytic activity whereas soleus is rich in type I fibers and presents higher mitochondrial content and oxidative capacity (Cornachione et al., 2011). Results from other studies have also shown distinct sensitivities of different muscles to statins (Waclawik et al., 1993) and insensitivity of soleus to these drugs (Schaefer et al., 2004).

Taken together, the present results provide evidence that chronic pravastatin administration to a murine model of familial hypercholesterolemia promotes mitochondrial dysfunctions in plantaris muscle that can be counteracted by antioxidants administered either *in vitro* (CoQ_10_) or *in vivo* (creatine). Therefore, we propose that inhibition of muscle mitochondrial respiration by pravastatin leads to an oxidative stress that in the presence of calcium opens the PTP. This mitochondrial oxidative stress caused by statin treatment also signals for cellular antioxidant system responses such as catalase upregulation.

## Author Contributions

EB performed the experiments, data analyses and interpretation and wrote the manuscript. AM and NL helped with the experiments, data analyses and interpretation. DdO and RC performed the lipidomic analyses and interpretation. HO and AV designed the work, interpreted the data, and wrote the manuscript.

## Conflict of Interest Statement

The authors declare that the research was conducted in the absence of any commercial or financial relationships that could be construed as a potential conflict of interest.

## Supplementary Material

FIGURE S1 **Protein oxidative markers evaluated in plantaris muscle of *LDLr^-/-^* mice. Aconitase activity (A)** and sulfhydryl content **(B)** were evaluated in plantaris muscle homogenates of control- and pravastatin- treated (40 mg/kg/day) *LDLr^-/-^* mice. Values are means ± standard deviation and are expressed as nmol/mg protein (One-Way ANOVA and Student’s *t*-test, non-significant). *N* = 6 for aconitase activity and *N* = 8 for sulfhydryl content.
