# Supplementary figures and images for: Pravastatin Chronic Treatment Sensitizes Hypercholesterolemic Mice Muscle to Mitochondrial Permeability Transition: Protection by Creatine or Coenzyme Q10
Source: Front Pharmacol. 2017 Apr 5;8:185. doi: 10.3389/fphar.2017.00185 (PMC5380726; doi:10.3389/fphar.2017.00185)

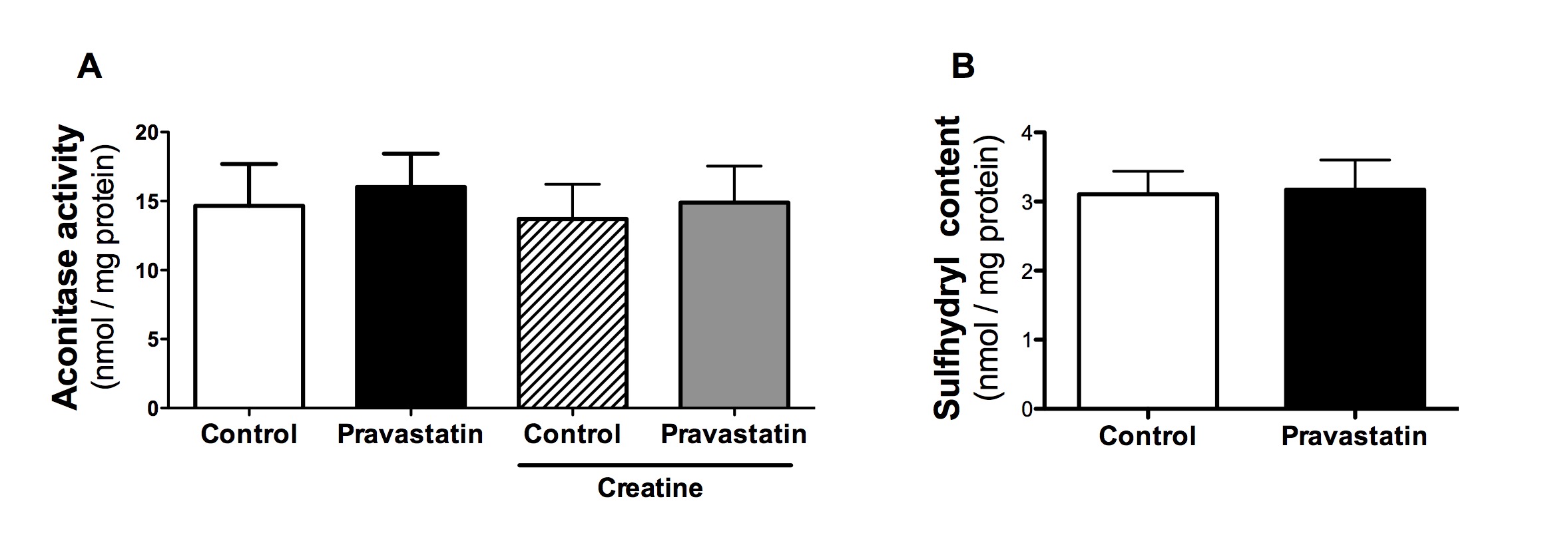

Supplement: FIGURE S1 — Protein oxidative markers evaluated in plantaris muscle of LDLr-/- mice. Aconitase activity (A) and sulfhydryl content (B) were evaluated in plantaris muscle homogenates of control- and pravastatin- treated (40 mg/kg/day) LDLr-/- mice. Values are means ± standard deviation and are expressed as nmol/mg protein (One-Way ANOVA and Student’s t-test, non-significant). N = 6 for aconitase activity and N = 8 for sulfhydryl content. [file Image_1.JPEG]
